# Synthesis and Application Dichalcogenides as Radical Reagents with Photochemical Technology

**DOI:** 10.3390/molecules28041998

**Published:** 2023-02-20

**Authors:** Cairong Wang, Yan Zhang, Kai Sun, Tingting Yu, Fei Liu, Xin Wang

**Affiliations:** 1 Department of Chemistry, Changzhi University, Changzhi 046011, China; 2 College of Chemistry and Chemical Engineering, Yantai University, Yantai 264005, China

**Keywords:** visible light, free radicals, diselenide, disulfide

## Abstract

Dichalcogenides (disulfides and diselenides), as reactants for organic transformations, are important and widely used because of their potential to react with nucleophiles, electrophilic reagents, and radical precursors. In recent years, in combination with photochemical technology, the application of dichalcogenides as stable radical reagents has opened up a new route to the synthesis of various sulfur- and selenium-containing compounds. In this paper, synthetic strategies for disulfides and diselenides and their applications with photochemical technology are reviewed: (i) Cyclization of dichalcogenides with alkenes and alkynes; (ii) direct selenylation/sulfuration of C−H/C−C/C−N bonds; (iii) visible-light-enabled seleno- and sulfur-bifunctionalization of alkenes/alkynes; and (iv) Direct construction of the C(sp)–S bond. In addition, the scopes, limitations, and mechanisms of some reactions are also described.

## 1. Introduction

Organosulfur and organoselenium compounds play an increasingly important role in organic synthesis and material science as well as in pharmaceutical applications [1]. In particular, C–S bonds exist in numerous natural products and biological and pharmaceutical compounds, such as nelfinavir, esomeprazole, and cephalosporin-type antibiotics, most of which have demonstrated antibacterial, antiviral, antitumor, and anti-inflammatory activities [2]. Furthermore, various organoselenium compounds have been identified as therapeutic agents, with anticancer, antiviral, and anti-Alzheimer’s properties [3]. They have also been explored as fluorescent probes, catalysts in organic synthesis, and functional organic materials [4]. Thus, the efficient construction of C–S and C–Se bonds has generated considerable interest in organic synthesis in the past decades.

The disulfide bond is an important functional group, existing in a large number of chemical and biological molecules, such as oxidized glutathione (GSSG), some proteins (thrombospondin-1 and protein disulfide isomerase [PDI]), and even some natural drugs (mitomycin disulfides and leinamycin). Disulfide bonds play a vital role in organisms and are generally obtained by covalently crosslinking two cysteine residues. GSSG and reduced glutathione (GSH) can maintain a balance of oxidation and reduction in cells, while disulfide bonds predominantly maintain the stability of protein conformation to ensure biological activity [5]. Compared with sulfur, Se has a larger diameter and a weaker negative charge [6]. The diselenide bond (Se–Se) is more redox active than the S–S bond in the microenvironment of cancer cells because its bond energy is lower than that of the disulfide bond (Se – Se = 172 kJ/mol; S – S = 268 kJ/mol) [7]. In addition, Sun et al. [8] studied the bond angles of sulfur bonds and selenium bonds and found that S–S (94.6°/97.9°) and Se–Se (89.9°/93.3°) had bond angles closest to 90°. In comparison, the diselenide bond has sufficient structural flexibility to establish the most favorable conformation during self-assembly.

As a green and sustainable energy source, visible-light photocatalysis has received increasing attention in synthetic chemistry. Indeed, lots of breakthroughs have been made through the effective fusion of radical chemistry and photocatalysis strategies [9]. In recent years, many visible-light-enabled direct reactions, including C−H selenylation/sulfuration and cyclization of diselenides/disulfides with alkenes and alkynes, among others, have been explored and successfully applied to the synthesis of various sulfur and selenium-containing compounds, showing outstanding synthetic value and potential applications. Although there are several other reviews on dichalcogenides, none of these reviews mainly focused on the application of dichalcogenides as radical reagents. Especially, the disulfide bond (S–S) and the diselenide bond (Se–Se) are very important and amazing. Along with the development of photochemical technology, in this review, we describe a recently established visible-light-induced synthesis strategy for sulfur and selenium-containing compounds with dichalcogenide (disulfides and diselenides) radical precursors. In addition, the reaction scope, application, and mechanism for some of these methods are also discussed. Finally, a number of issues and challenges are explored, such as the need for more catalytic approaches that should be explored with electrochemical strategies and a migration cyclization strategy involving disulfide and diselenide as radical reagents that should also be established. 

## 2. Synthesis of Dichalcogenides (Disulfides and Diselenides)

Disulfides and diselenides have interesting properties and are also widely used as versatile free radical reagents in many organic transformations. For these reasons, chemists have made considerable efforts to design and develop new and effective methods for the construction of disulfides and diselenides. However, a literature review indicates that synthetic methods for disulfides and diselenides are limited. Here, we briefly summarize some well-known synthetic strategies based on the precursors used to generate disulfides and diselenides. Each of these reactions will be discussed in summary in the following introduction.

**Figure 1:**
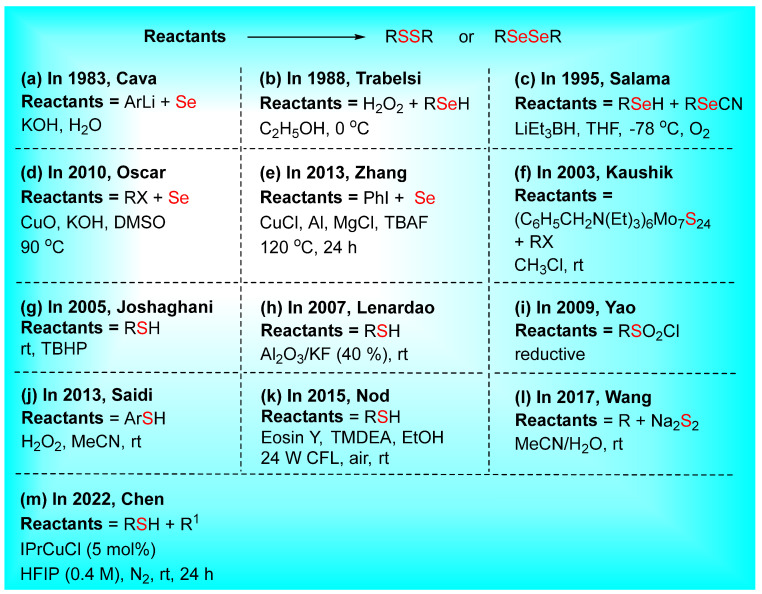
Summary of recent synthetic strategies for disulfides and diselenides.

Lithium reagent method (Figure 1a): In 1983, Cava [10] improved the lithium reagent method by using *N*,*N*-dialkyl-thiocarbamoyl chloride, avoiding the by-product of diaryl-monoselenide in the original method. The yield using this approach was improved from 75% to 90%, but this method is carried out at a low temperature (−78 °C), while the preparation of the reagents required for the synthesis of diselenide is also complex.

Selenol oxidation method (Figure 1b): In 1988, Krief [11] discovered that diselenides can be prepared rapidly and in large quantities by using H_2_O_2_ or the corresponding selenic acid as the oxidant. Recently, Chinese scholars have found that diselenides can also be efficiently prepared using trichlorocyanuric acid [12].

Selenium transfer reagent method (Figure 1c): In 1995, Zhou et al. [13] first reported the use of a selenium transfer reagent to synthesize diselenide. Under mild conditions, the reaction of selenoamides with various alkyl halogenated hydrocarbons can produce alkyl diselenides in high yields. The disadvantage of this method is that selenoamides are not easy to prepare.

Catalytic cross-coupling method (Figure 1d): In 2010, Oscai’s group [14] first reported the preparation of diselenide compounds via cross-coupling catalyzed by copper oxide nanoparticles. Compared with traditional methods, nanocatalysts have been effectively processed, with their high surface area and special morphology, making them effective as catalysts in organic synthesis. This method is applicable not only to the preparation of alkyl and aryl diselenides but also to heterocyclic diselenides, with high universality and generally high yields.

Copper-catalyzed one-pot method (Figure 1e): In 2013, Zhang et al. [15] developed a method using cuprous chloride as the catalyst, acetylacetone as a ligand, and an aromatic halide as the raw material, which was directly reacted with selenium to synthesize diaryl selenide compounds.

Sulfur reagent transfer method (Figure 1f): In 2003, Kaushik et al. [16] also reported a new and effective sulfur transfer reagent (C_6_H_5_CH_2_N(Et)_3_)_6_-Mo_7_S_24_) to react with halogenated hydrocarbons to obtain disulfides. The reaction conditions are mild, and the yield is satisfactory.

Oxidative coupling method (Figure 1g): In 2005, Joshaghani [17] reported a new method for the mass production of disulfides. This method uses tert-Butyl hydroperoxide (TBHP) as an oxidant to directly transform thiols to disulfides, which is fast and efficient.

Microwave irradiation method (Figure 1h): In 2007, Lenardao’s group [18] discovered that symmetric disulfides were rapidly synthesized from mercaptans under solvent-free conditions using a solid Al_2_O_3_/KF system as a catalyst under microwave irradiation at room temperature. This improved method is generally aimed at the conversion of liquid mercaptans to disulfides, and the yields range from moderate to good. In addition, the catalyst system could be used twice without any decrease in activity.

Reductive coupling method (Figure 1i): In 2009, Yao [19] reported that triphenylphosphine was used for the reductive coupling of benzenesulfonyl chloride at room temperature to easily obtain disulfides.

Nanocatalysis method (Figure 1j): In 2013, Saidi [20] found that using nano iron oxide as a catalyst and hydrogen peroxide as an oxidant at room temperature can efficiently oxidize mercaptan to disulfide ether. Nano iron oxide is an efficient and recyclable catalyst, and hydrogen peroxide is a green oxidant. The advantage of this method is that the catalyst is stable and reliable, the reaction conditions are mild, and the selectivity is high.

Oxidation of thiols (Figure 1k): In 2015, Noël et al. [21] used eosin Y dye to oxidize thiols to disulfide derivatives under visible light. Compared with heavy metal catalysts, using organic dyes as photosensitizers is less toxic and less expensive. This method is suitable for aliphatic thiols, aromatic thiols, and heterocyclic thiols, with yields ranging from 86% to 99%. The advantage of this method is that the reaction time can be reduced from hours to minutes. Moreover, drugs such as oxytocin and disulfiram can be synthesized using this method.

Conversion from Na_2_S_2_ (Figure 1l): This method is used to prepare symmetrical disulfide derivatives by the reaction of alkyl bromide with Na_2_S_2_. In 2017, Wang and colleagues [22] reported a method for the synthesis of symmetric disulfides. This method uses a bromine-containing dienamine reacted with Na_2_S_2_ to afford the corresponding symmetrical disulfides in 54% yield.

Recently, Chen’s group [23] reported a class of N-acyl hypersulfonamides containing S–N bonds based on copper-catalyzed amination of azobene-mediated thiols. This class of S–N compounds was shown to be a good thiol sulfide reagent for the preparation of various disulfides (Figure 1m).

## 3. Application of Dichalcogenides under Visible-Light Irradiation

### 3.1. Cyclization of Diselenides/Disulfides with Alkenes and Alkynes

#### 3.1.1. Cyclization of Diselenides with Alkenes and Alkynes

The ingenious design of novel radical addition cyclizations has emerged as an increasingly powerful tactic to construct complex molecules in an atom- and step-economical manner. Over the past decade, visible-light-driven photoredox catalysis has stimulated a resurgence of interest in the exploration of radical reactions.

**Scheme 1:**
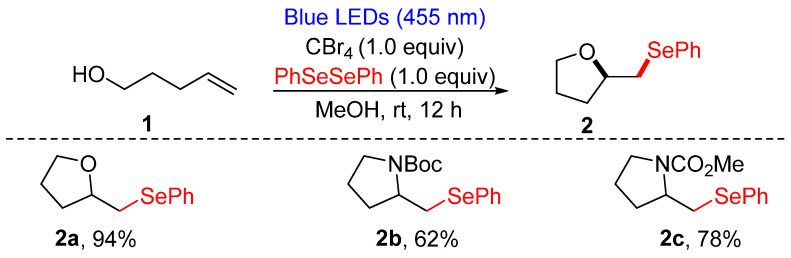
Visible-light-promoted synthesis of *β*-selenyl *O*-heterocycles and *N*-heterocycles.

In 2013, Ragains and coworkers reported a visible-light-promoted selenocyclization of alkenes at room temperature (Scheme 1) [24]. In this reaction, bench-stable PhSeSePh was combined with CBr_4_ under the irradiation of a 5W blue light-emitting diode (LED), resulting in the in situ generation of reactive PhSeBr. This reaction showed a broad substrate scope, generating *O*-heterocycles in high yields along with *N*-heterocycles in moderate to high yields. Notably, in CH_2_Cl_2_ as a solvent, diphenyl ditelluride provided successful tellurofunctionalization products in 53–75% yields. To further demonstrate the application of this method, the amaryllidaceae alkaloid, *γ*-lycorane, was synthesized. Free radical validation experiment and density functional theory (DFT) calculation suggested visible-light irradiation promoted the phenylselenyl radical abstraction of bromine from CBr_4_ to generate phenylselenyl bromide in situ. The detailed mechanism of these reactions is still under investigation.

**Scheme 2:**
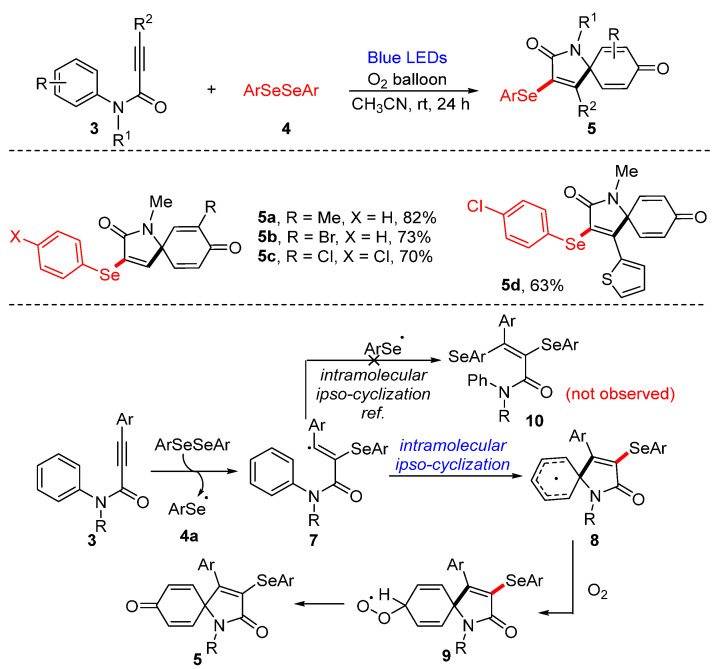
Visible-light-promoted synthesis of spiro [4,5] trienones.

In 2017, an efficient photocatalyst-free approach for the preparation of selenium-substituted spiro [4,5] trienones based on visible-light-induced selenium radical cyclization of *N*-aryl alkynamides was described by Baidya and coworkers (Scheme 2) [25]. Diverse *N*-aryl alkynamides and diaryl diselenides bearing electron-donating as well as electron-withdrawing groups in an aryl ring achieved the products under an oxygen atmosphere at room temperature. In addition, good yields were achieved in gram-scale reactions. A spiro-ring-opening strategy was realized to give fully substituted acryl amides **5**.

To probe the reaction mechanism, control experiments were performed. The reaction yields dropped significantly in the presence of radical scavengers such as butylated hydroxytoluene (BHT) and 2,2,6,6-tetramethylpiperidinyl 1-oxide (TEMPO), and a plausible reaction mechanism was proposed, as shown in Scheme 2. First, under visible-light irradiation, the diselenide is homolytically cleaved to produce a selenium radical **4a**, which subsequently attacks the triple bond position of compound **3** to obtain radical intermediate **7**. The final radical **7** is obtained as intermediate **8** by intramolecular cyclization. The intermediate **8** undergoes an oxidative de-aromatization reaction in an oxygen environment to obtain the desired product **5**.

**Scheme 3:**
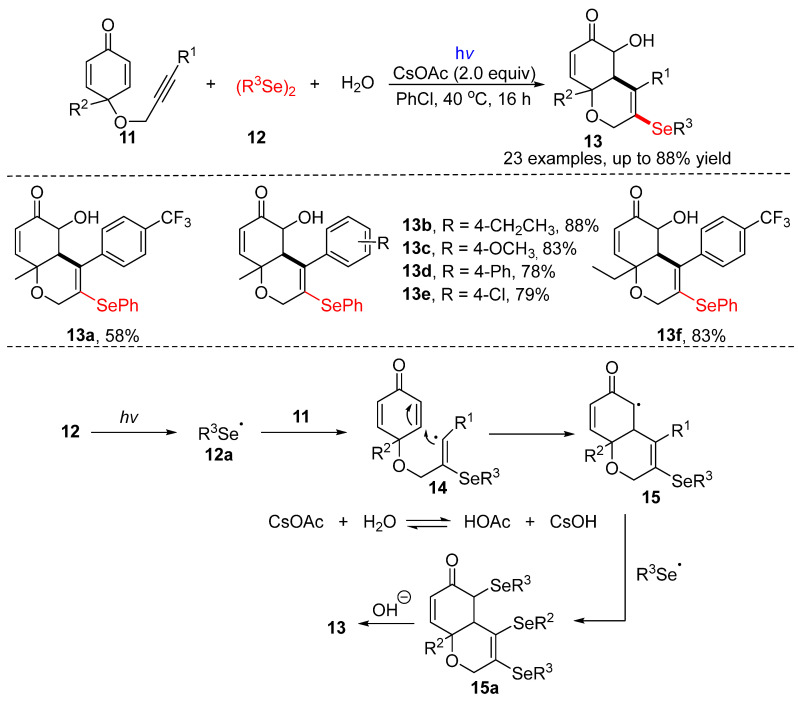
Visible-light-induced cascade reaction of alkyne-tethered cyclohexadienones and diselenides.

In 2019, Xu and coworkers reported an Se radical-triggered multi-component tandem cyclization of alkyne-tethered cyclohexadienones **11** and diaryl diselenides **12** under the irradiation of 25 W white LEDs at 40 °C (Scheme 3) [26]. The cascade cyclization starts with the addition of selenyl radical **12a** to the alkyne, generating a vinylic radical **14**, and proceeds in a 5-*exo*-trig fashion to **15**, with the formation of the C−C bond. The intermediate **15** captures another selenyl radical, **12a**, yielding bis-selenyl chromenones **15a**. In the presence of water and a base, the final step of nucleophilic substitution affords compound **13**. Therefore, in the absence of a base, the authors obtained the bis-selenyl derivatives **15a**.

**Scheme 4:**
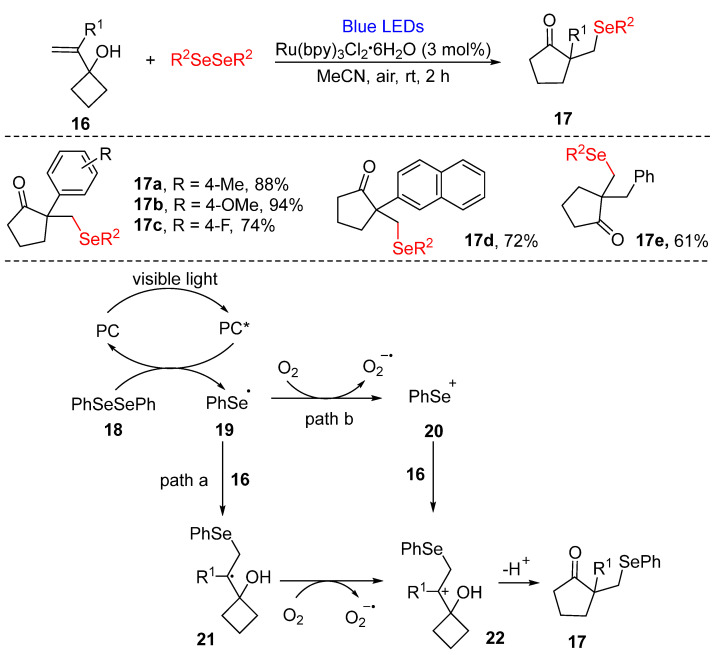
Ru (II) catalyzed, photochemical synthesis of *β*-organoselenyl cyclopentanones.

In the same year, Kim et al. developed a practical method to synthesize selenated cyclopentanone derivatives **17** by photooxidation-catalyzed selenation through a ring expansion reaction with diselenides and alkenyl cyclobutanol derivatives (Scheme 4) [27]. A simple method was provided for the preparation of selenated cyclopentanone derivatives. The reaction occurred in acetonitrile solvent and was irradiated with blue light in air for 11 h. Ru(bpy)_3_Cl_2**·**_6H_2_O was used as a photocatalyst, with a yield of up to 94%. The authors performed a series of relevant control experiments to verify the mechanism of this reaction. The report described how the formation of the product was significantly inhibited by the addition of the radical scavenger TEMPO under standard conditions, demonstrating that the transformation involved a radical pathway. The authors proposed the reaction mechanism shown in Scheme 4. For example, the photocatalyst Ru(bpy)_3_Cl_2**·**_6H_2_O is converted to the excited state [Ru(bpy)_3_Cl_2**·**_6H_2_O]* under visible-light irradiation. Afterwards, the selenium radical **19** reacts with 1-(1-phenylvinyl)cyclobutanol **16** to give the carbon radical **21**, which is oxidized by oxygen to the cationic intermediate **22** (path a). By 1,2-alkyl migration of intermediate **21**, the alcohol rearranges to form cyclopentanone **17**. Alternatively, radical **19** can be oxidized by oxygen to the selenium cation **20**, which reacts with 1-(1-phenylethenyl)cyclobutanol **16** to give the corresponding cation **22** (path b).

**Scheme 5:**
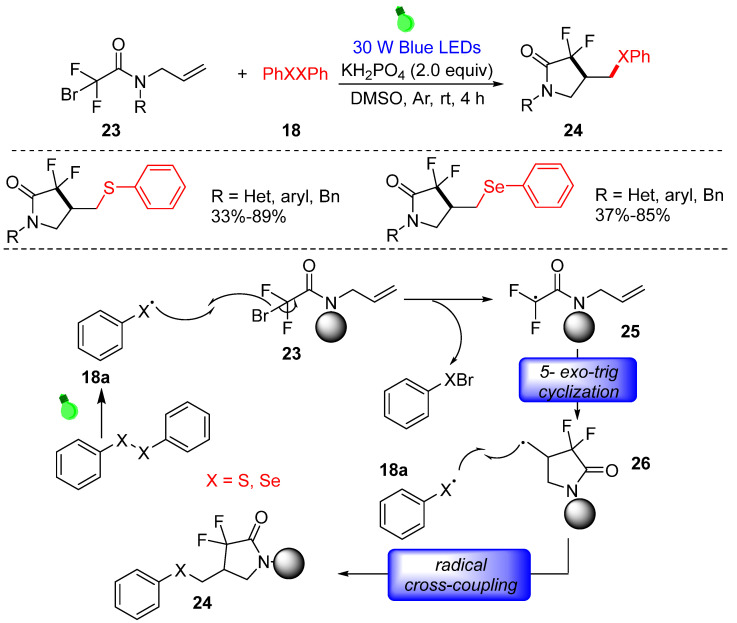
Visible-light-induced cross-coupling cyclization.

In 2020, the Yang group disclosed a visible-light-induced, catalyst-free, and metal-free method for the construction of 4-sulfo/seleno-*α*,*α*-difluoro-*γ*-lactams via radical-initiated tandem cyclization (Scheme 5) [28]. In this process, **23** reacts smoothly with diphenyl disulfides under irradiation with 30 W blue LEDs, with K_2_HPO_4_ as a base to afford the desired products at room temperature. It is worth mentioning that the addition of Ru(bpy)_3_PF_6_ or Ir(ppy)_2_(dtbbpy)PF_6_ resulted in relatively lower yields of 39%. Control experiments demonstrated that visible light was vital for this transformation. Interestingly, for this photoinduced transformation, the 5-*exo*-trig cyclization dominated the process, and no 6-*endo*-trig cyclization product was isolated. This protocol exhibits good functional group tolerance and affords a variety of 4-thio- and 4-seleno-substituted 3,3-difluoro-*γ*-lactams in moderate to good yields. To gain insight into the mechanisms underlying this reaction, a series of control experiments were conducted. The addition of TEMPO suppressed the reaction and generated the addition compounds TEMPO−**23** and TEMPO−**18**. The results of emission quenching experiments indicated that it is impossible to form the corresponding electron–donor–acceptor complexes for substrates **23** and **18**. Based on these results, a feasible mechanism for this reaction was proposed (Scheme 5). Initially, the radicals Ph−X^•^ and Ph−XBr are generated under visible-light irradiation. The consumption of Ph–XBr by the base also promotes this transformation. The radical Ph–X^•^ then abstracts the bromine atom from substrate **23** to offer the radical intermediate **25**, which undergoes rapid 5-*exo*-trig cyclization to form the radical **26**. Ultimately, the radical **26** and Ph−X^•^ easily form the target **24** via radical–radical cross-coupling. Another radical pathway could not be completely excluded, as indicated by the combination of products detected.

**Scheme 6:**
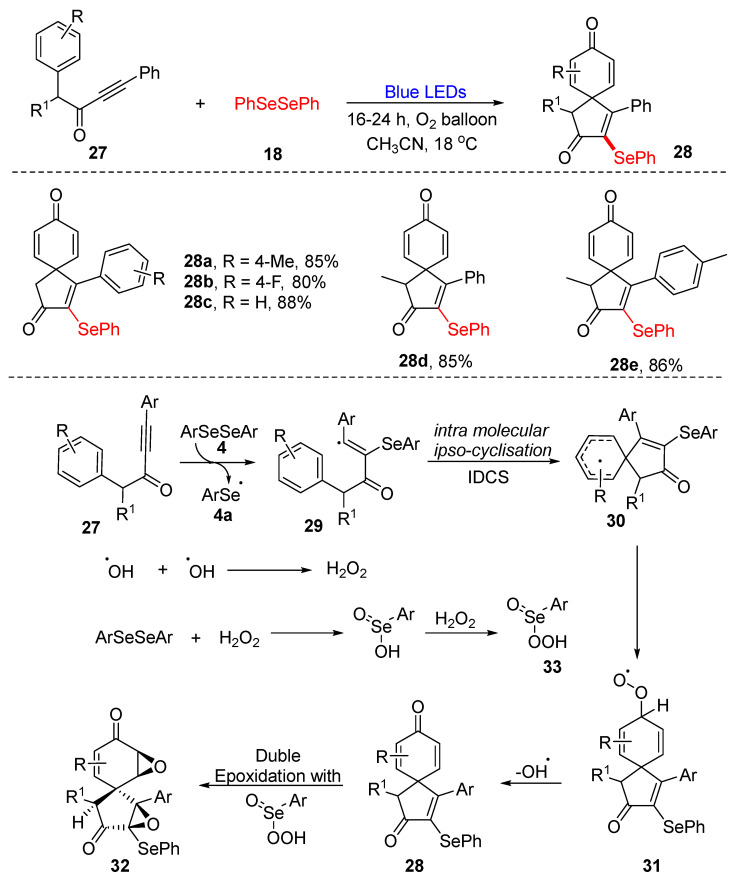
Spirocyclization of homologated-ynones.

In 2020, Reuter’s group [29] reported the visible-light-assisted dearomatic carbon selenide iodine cyclization of aromatic homologues (Scheme 6). At different temperatures, alkynones can deliver selenized cyclohexadienones and spiro diepoxides. The reaction mixture was exposed to blue LED illumination at 18 °C, using acetonitrile as solvent. Starting from 1,4-diphenylbut-3-yn-2-one **27** and diphenyl diselenides as the selenium source, selenospiro-cyclohexadienones were obtained using oxygen in up to 88% yield. In addition, experimental studies on free radical control confirmed that the process proceeds through the free radical pathway. The mechanism was rationally described based on control experiments and competition experiments, as shown in Scheme 6. Namely, the addition of aryl selenium radical **4a** produced under blue LED irradiation to the alkyne bond of **27** produces vinyl radicals **29**, which subsequently undergo intramolecular cyclization to give intermediate **30**. In an oxygen environment, intermediate **30** undergoes peroxidation and is converted to the peroxy intermediate **31**, which finally provides spirocyclized cyclic alkenones **28** together with an OH radical. Two OH radicals combine to deliver hydrogen peroxide followed by in situ generations of benzeneperoxyseleninic acid **33** in the presence of diaryl diselenide **4**, which functions as an epoxidizing agent. The spiro-cyclized cyclic enone **28** undergoes double epoxidation with the in situ-generated epoxidizing agent **33**, facilitating the epoxidation of the adjacent enones to the di-epoxide **32**.

**Scheme 7:**
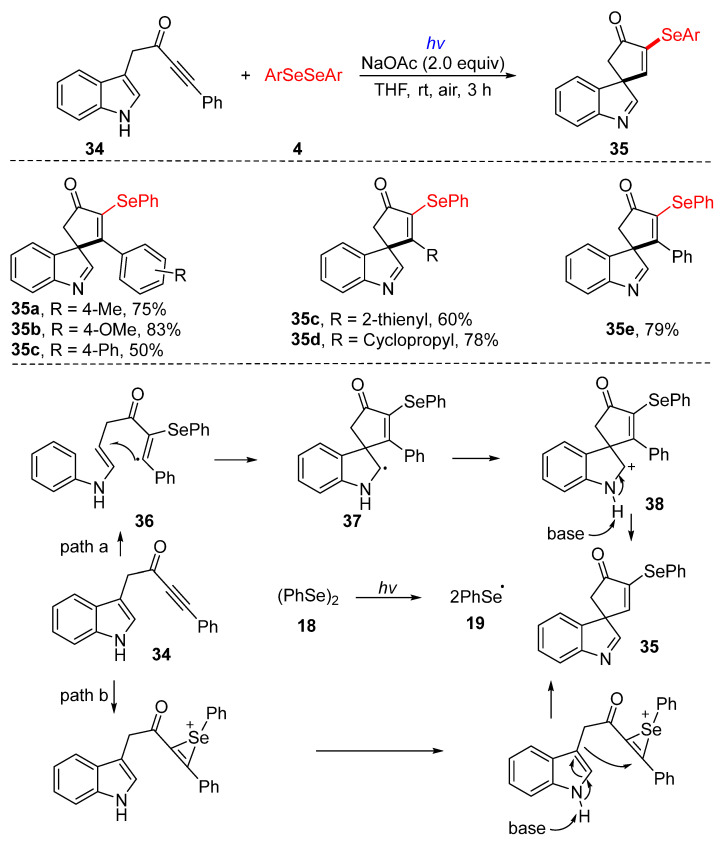
Visible-light-promoted synthesis of 3-selenospiroindolenines.

In 2020, Xu and coworkers further developed the visible-light-induced selenocyclization reaction of indolyl-ynones **30** with diselenides at room temperature under an air atmosphere (Scheme 7) [30]. Diverse 3-selenospiroindolenines bearing various functional groups were obtained in moderate to good yields. Similarly, a phenylselenyl free radical is generated from diphenyl diselenide under visible-light irradiation. The desired product is then obtained through the radical addition/oxidation/deprotonation pathway.

Based on free-radical verification experiments and previous literature reviews, Scheme 7 proposes and describes two possible pathways. The irradiation of diphenyl diselenide **18** produces phenylselenyl free radical **19**. In path a, the addition of indolyl-ynones **34** to the phenylselenyl radical **19** affords an alkenyl radical **36**, followed by cyclizing with the indole at its 3-position to form a spirocyclic radical intermediate **37**. Oxidation of **37** to **38** in air then undergoes dehydrogenation in base conditions to afford the desired product **35**. In path b, the reaction proceeds by oxidation of the phenylselenyl radical **19** to PhSe^+^ in air, which reacts with the alkyne group of indolyl-ynone **34**, leading to the formation of seleniumion. Then, cyclization at the 3-position of indole gives the desired product **35**.

**Scheme 8:**
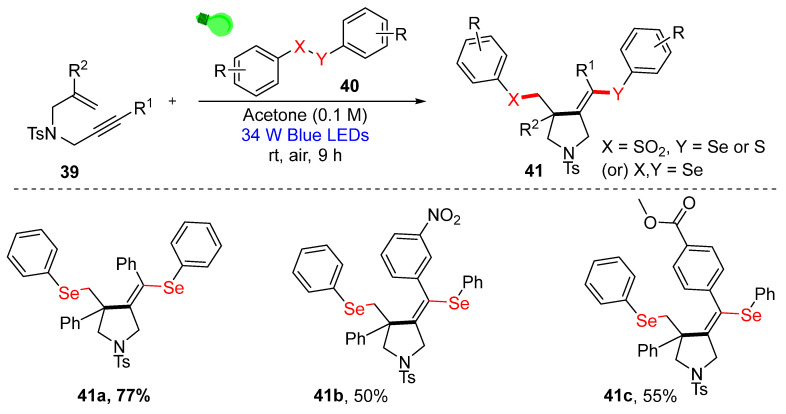
Visible-light-promoted synthesis of seleno-containing pyrrolidines.

Very recently, Wang disclosed a regio- and chemoselective radical cascade cyclization of 1,6-enynes **39** and areneselenosulfonates **40** under 34 W blue LED irradiation in the air without any photocatalysts (Scheme 8) [31]. Numerous substrates **39** were examined, and the corresponding cyclized products **41** were obtained in good to excellent yields. This reaction also proceeded smoothly using diaryldiselenides **40** with 1,6-enynes and produced the desired products with moderate to good yields. However, this method was not applicable when the chain length was increased from one to two or three. The internal alkene and free amine in enyne were also not tolerant of this transformation. This protocol offers an effective approach to building selenium-substituted pyrrolidine derivatives via multiple chemical bond constructions in a 5-*exo*-dig fashion, including one C–S bond, one C–Se bond, and one C–C bond.

**Scheme 9:**
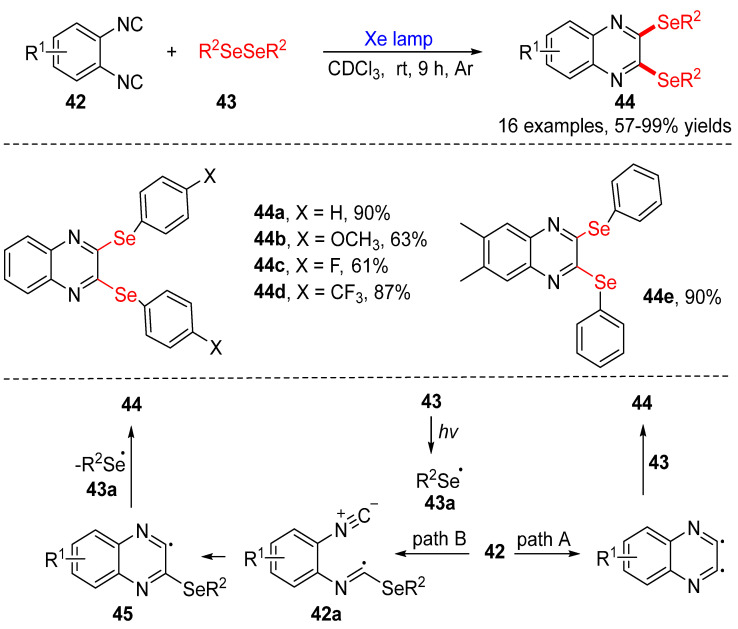
Photoinduced cyclizations of *o*-diisocyanoarenes with diselenides.

Notably, the cyclization reaction does not require photocatalysts or other additives. The bond energy of the Se–Se bond allows the production of photoinduced phase Se radicals and is further used in a wide range of reactions. In 2020, Tran and colleagues reported the synthesis of diselenyl quinoxalate **44** from *o*-diisocyanate arenes **42** and diaryl or dialkyl diselenides **43** with moderate to good yields (Scheme 9) [32].

The addition of the radical inhibitor TEMPO under the standard reaction suggested that the transformation involved a radical process. Control experiments also showed that diselenides were activated through a photocatalytic energy transfer pathway. Based on the results of relevant control experiments and previous references, two possible mechanisms have been depicted, as shown in Scheme 9. The first mechanism involves a photoinduced aza-Bergman-type cyclization followed by trapping of the 11 species (path A). In the second mechanism (path B), the R^2^Se radical species **43a** attacks the isocyanide group to form the imidoyl radical intermediate **42a**, followed by intramolecular addition to the vicinal isocyanide group. Finally, intermediate **45** reacts with another equivalent of diorganoyl diselenide, R^2^Se **43a**, affording the bis-selenylquinoxalines **44**.

**Scheme 10:**
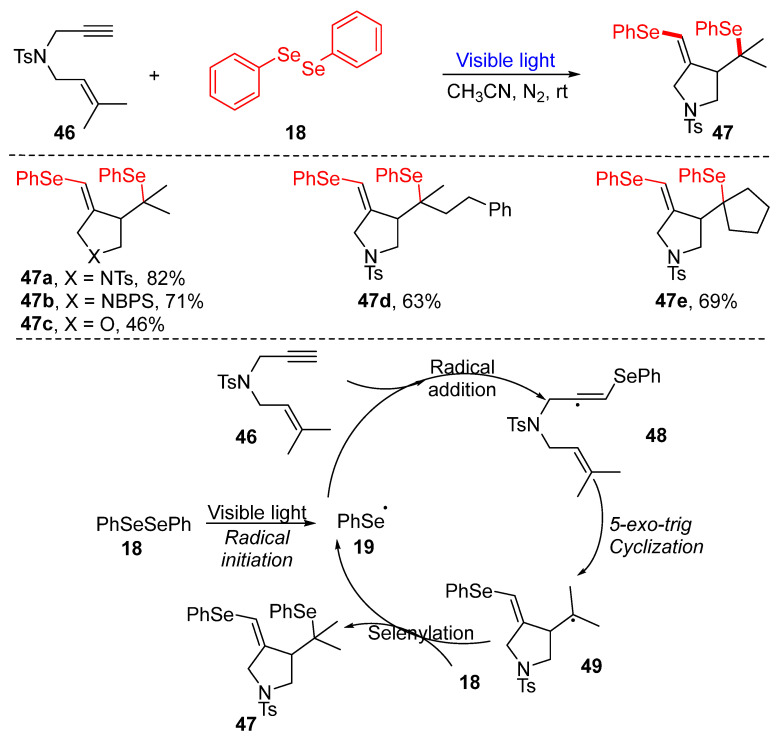
Photoinduced regio- and chemoselective radical cyclization of unactivated 1,6-enynes with selenides.

In 2021, Zhu and colleagues [33] described a visible-light-mediated diaryl selenide cyclization of 1,6-enylene **46** with diselenides ether radical cyclization (Scheme 10). It is proved that terminal alkyne and inner alkyne derived from 1,6-enylene are suitable for this synthesis, and the cyclized product **47** with a good yield is obtained. Through validation experiments based on free radicals, the mechanism of radical cyclization is proposed. First, diaryl diethers **18** produce aryl selenium radicals **19** under visible-light irradiation. Then, a selenium free radical **19** is added to the carbon–carbon triple bond of alkyne **46** to form a carbon radical intermediate **48**. Then the intermediate vinyl free radical **48** undergoes an intramolecular 5-external triangular cyclization reaction to generate a tertiary carbon-alkyl radical **49**. Finally, the diselenium compound is captured by radical **49** to obtain the target product **47** and the aryl selenyl radical **19**.

#### 3.1.2. Cyclization of Disulfides with Alkenes and Alkynes

**Scheme 11:**
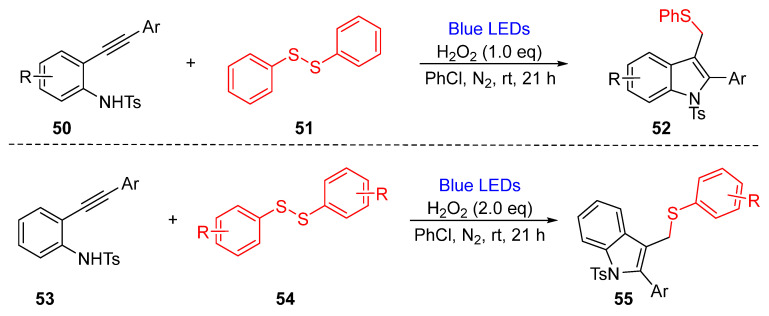
Synthesis of benzo heterocycles.

In 2017, Wang’s group [34] developed a simple and effective scheme for the preparation of indoles using H_2_O_2_ and visible light to drive the reaction of 2-alkynyl aniline with disulfides. The corresponding products were synthesized at room temperature with high yields and without additional conditions. In the same year, another method was published for the preparation of benzothiophenes, which was achieved by visible-light induction of *o*-alkynyl anilines and disulfides without the addition of metals or photocatalysts (Scheme 11). The reaction offers good yields, broad substrate expansion, and simple operating conditions.

**Scheme 12:**
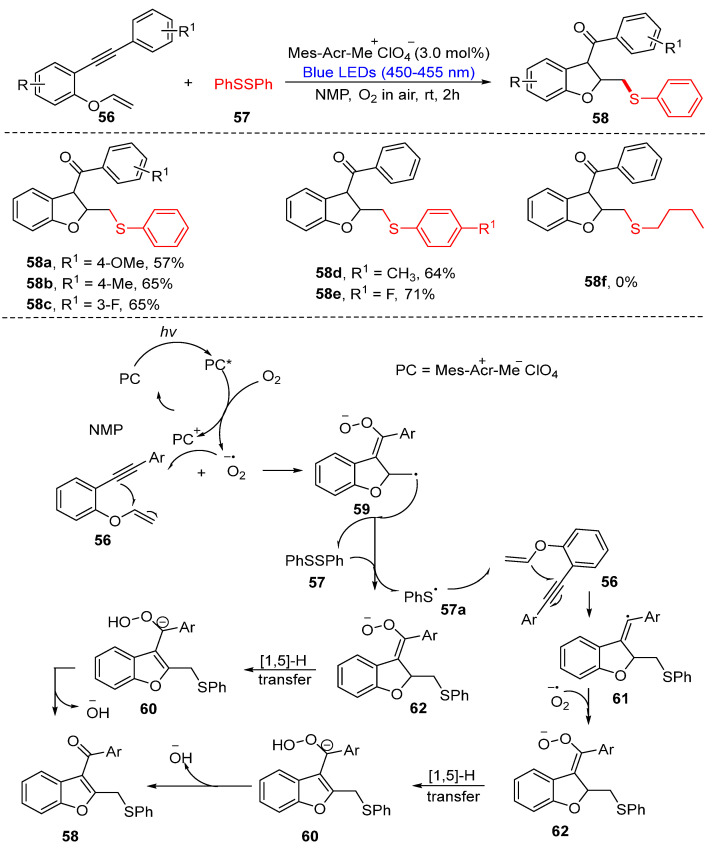
Synthesis of benzofurans.

Benzofuran-like scaffolds are usually found in natural products and display biological and pharmaceutical activity. In 2021, Li’s group [35] developed a simple and effective method for the visible-light-induced tandem cyclization of 1,6-benzenes with disulfides for the synthesis of functionalized benzofurans (Scheme 12). 

The reaction proceeded smoothly under metal-free conditions in room-temperature air, providing the desired products with broad functional group tolerance and good yields. On the basis of experiments verified by radicals, the authors propose a plausible mechanism, as shown in Scheme 12. On the one hand, PC (Mes-Acr-Me-ClO_4_) is transformed into the excited state PC* under visible-light irradiation, which is subsequently oxidized by oxygen to PC^+^ while generating superoxide radical anions [36]. In the presence of N-methyl-2-pyrrolidone (NMP), PC^+^ again becomes PC in the catalytic cycle. On the other hand, the superoxide radical anion is added to the alkyne **56** to provide the alkyne radical anion **59**, which reacts with diphenyl disulfide **57** to produce the peroxyalkynyl sulfide intermediate **62** and release the sulfur group (PhS^•^). The resulting **62** then undergoes a [1,5]-hydrogen transfer to give intermediate **60**, which then loses the hydroxyl anion to yield the desired product **58**. Moreover, the formed phenylthio group (PhS^•^) can be added to another substrate molecule **56** to give vinyl **61**, which reacts with the superoxide radical anion to produce intermediate **62**. Intermediate **62** repeats the [1,5]-hydrogen transfer and elimination of the hydroxyl anion (HO^-^) to give the corresponding product **58**.

### 3.2. Direct Selenylation/Sulfuration of C−H/C−C/C−N Bonds

#### 3.2.1. Direct Selenylation/Sulfuration of C−H Bonds

**Scheme 13:**
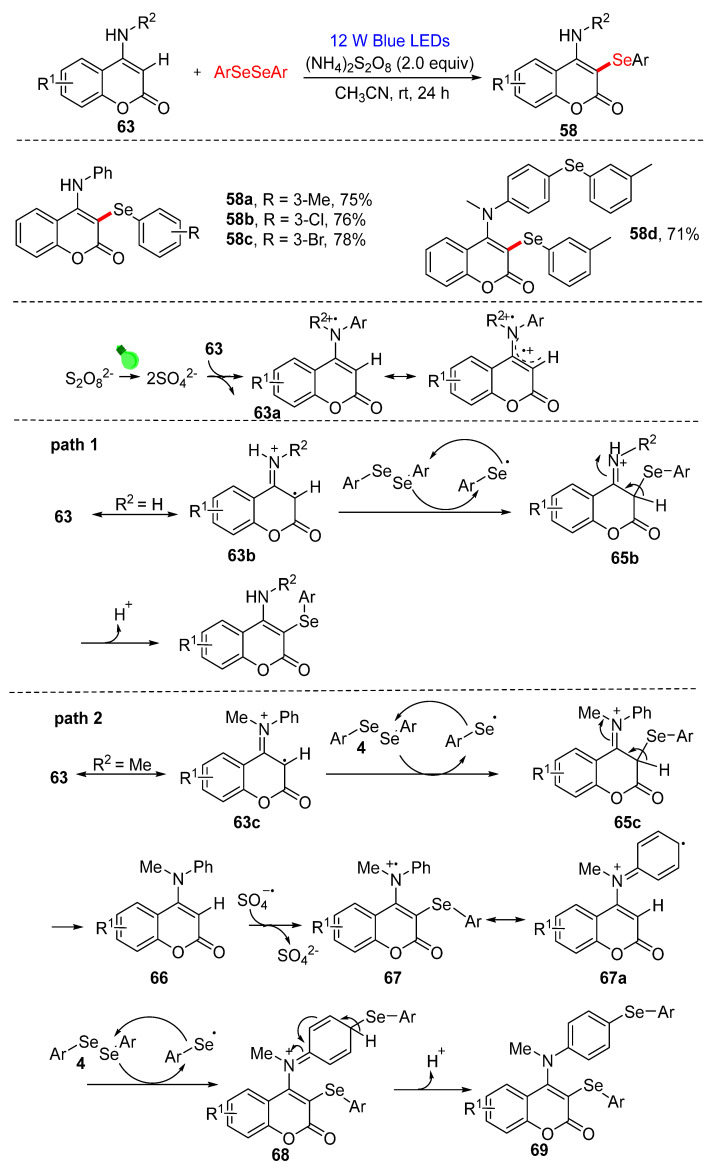
(NH_4_)S_2_O_8_ mediated photoinduced selenylation coumarins.

Coumarins form a well-known class of naturally occurring heteroarenes, which exhibit a diverse range of biological and medicinal activity, including anticancer, antibacterial, and anticoagulant properties [37]. Their use in materials science is also well established [38]. In 2018, Yang and colleagues [39] reported the photoinduced C(sp^2^)−H selenium functionalization of 4-amino-substituted coumarins **63** in the presence of ammonium persulfate (Scheme 13). Under optimal conditions, various 4-amino-substituted coumarin derivatives were found to be converted to the desired products with diselenides in moderate to good yields; unfortunately, 2*H*-chromen-2-one is not suitable for this visible-light-induced selenization reaction.

Although the mechanism of the visible-light-induced selenization pathway remains unclear, Scheme 13 proposes two possible routes based on radical verification experiments and previous literature reports [40,41,42]. First, (NH_4_)_2_S_2_O_8_ is irradiated with visible light to give the reactive radical anion SO_4_^•−^. Then, a single-electron transfer process occurs between **63** and SO_4_^•−^ to give the corresponding radical intermediate **63a**. In path 1, intermediate **63b** reacts with diselenide **4** to form intermediate **65b**. In path 2, intermediate **63c** reacts with diselenide **4** to form the C−3 selenide product **65c**. A single-electron transfer process occurs between **66** and SO_4_^•−^ to give the corresponding radical cation **69**.

**Scheme 14:**
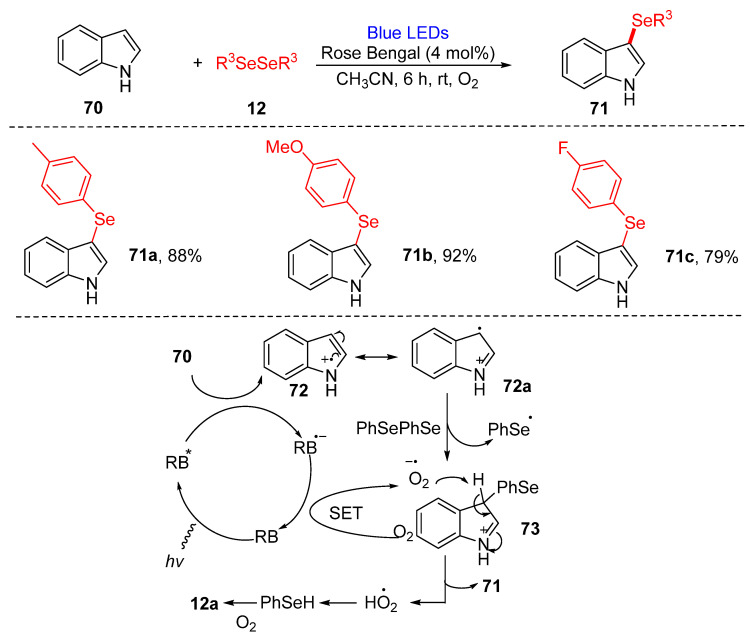
RB catalyzed photochemical selenylation of indoles and (hetero)arenes.

The use of inexpensive and readily available organic dyes as photocatalysts has attracted extensive attention in the synthesis community [43]. In 2018, Braga and colleagues reported a more environmentally friendly, photoinduced synthesis of selenium aromatic compounds **71** (Scheme 14). Under visible-light irradiation, using rose bengal (RB) as the photocatalyst and air as the terminal oxidant, the indole was selenized with a half molar equivalent of diselenide to obtain a selenium-containing indole compound in good yield. The reaction has broad functional group tolerance and is also applicable to a variety of heterocycles, including indazole, imidazopyridine, imidazolyl, and imidazothiazole, as well as aniline, anisole, *β*-naphthylamine, and *β*-naphthol, for example. Validation experiments based on free radicals allowed the authors to propose a possible reaction mechanism. First, RB changes from the ground state to the excited state RB^*^ under visible-light irradiation, and **70** undergoes single-electron transfer to form radical cation **72**. **72** obtains resonance formula **72a** through resonance and reacts with diselenide to form intermediate **73** in the presence of O_2._ Next, intermediate **73** loses a proton at C−3, yielding the corresponding 3-selenoindole **71**. At the same time, the remaining selenium radicals are oxidized back to diselenide **12a**, entering the second cycle.

**Scheme 15:**
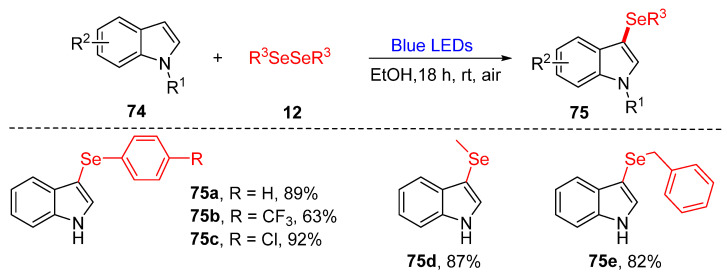
Blue LEDs induced C(sp^2^)−H bond selenylation of indoles and activated arenes.

In 2019, Lemir’s group published a method for synthesizing 3-selenoindoles from diselenides and indoles or electron-rich olefins as raw materials and ethanol as a green solvent (Scheme 15) [44]. By this simple and environmentally friendly method, several 3-selenoindoles and many asymmetric diselenides can be obtained in high yields. In contrast, indoles with electron-withdrawing groups do not react.

**Scheme 16:**
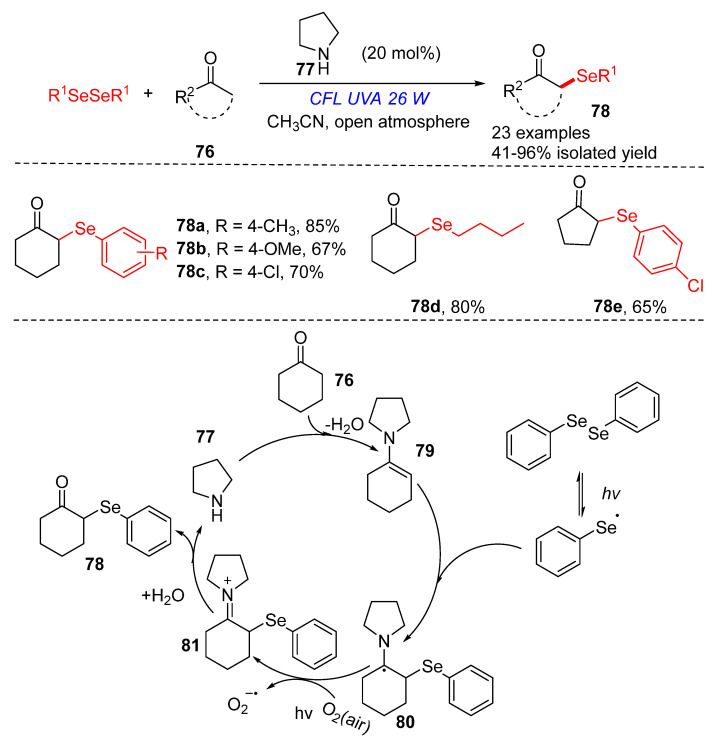
Photoinduced metal-free *α*-selenylation of ketones.

Recently, *α*-selenoketones have attracted attention due to their versatile applications in organic synthesis as well as their biological activity in the treatment of disorders such as depression and anxiety [45]. Most of the *α*-selenofunctionalization protocols include the in situ generation of nucleophilic selenium reagents or previous formation of an electrophilic species from diselenides. In 2020, Schneider [46] reported an efficient photoinduced *α*-selenide reaction of ketones without metal additives or photosensitizers, providing a green scheme for the synthesis of various *α*-selenide ketones using light energy (Scheme 16). In this experiment, *α*-selenide ketones were prepared using equal amounts of diselenides and alkyl ketones **76** under compact florescent lamp ultraviolet A (CFL UVA) irradiation at 26 W for 6 h with 20 mol% pyrrolidine as the organocatalyst and CH_3_CN as solvent. Subsequently, the universality of the substrate was explored, and it was found that when the diorganodiselenide and the cyclic and acyclic ketones had different substituents, the reaction could proceed smoothly to create a series of *α*-selenide products with moderate to excellent yields. These findings demonstrate the generality of the method.

The addition of the radical inhibitor TEMPO to the standard reaction suggests that the transformation involves a radical process. Control experiments also showed that diselenide is activated through a photocatalytic energy transfer pathway. Based on the results of relevant control experiments and previous references, a possible mechanism was depicted, as shown in Scheme 16. First, **79** is formed in situ by the condensation of pyrrolidine **77** with cyclohexanone **76**. Then, diselenide is cleaved to selenium radicals by Se−Se bonding under visible-light irradiation. The selenium radical undergoes addition to the enamine **79** to form the selenated intermediate **80**. Subsequently, the species **80** can be oxidized by atmospheric O_2_ to the imino cation **81**, providing the desired product **78** and regenerating the organocatalyst **77**.

**Scheme 17:**
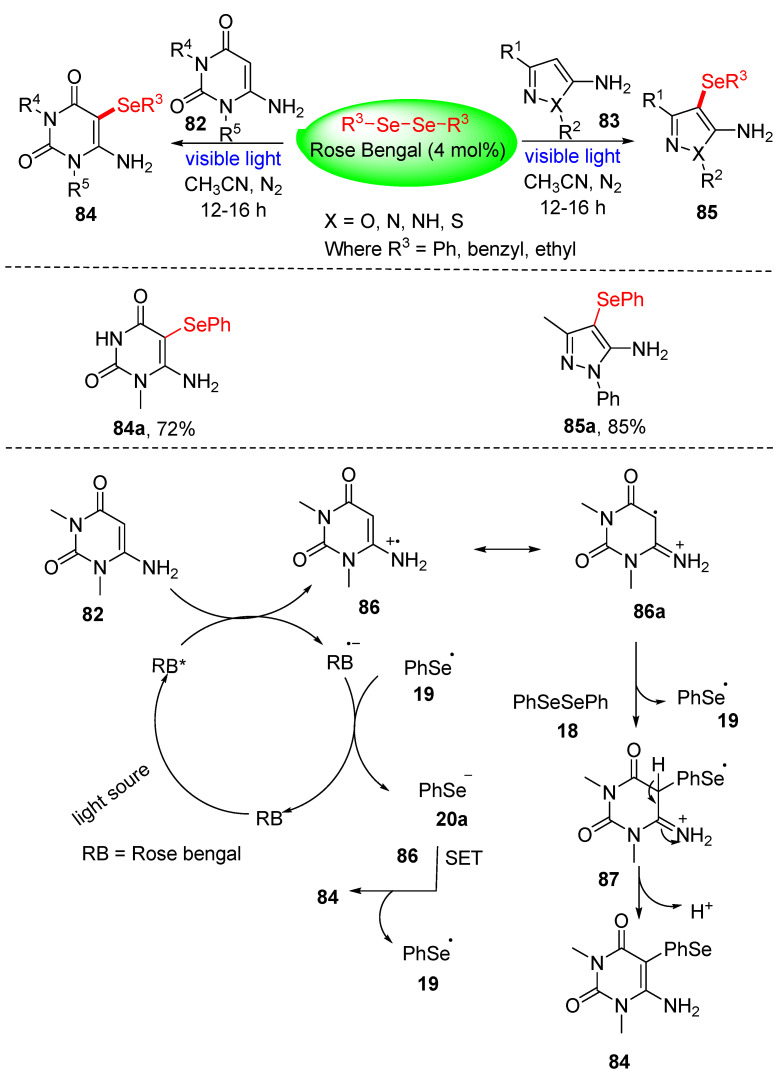
Visible-light-mediated C(sp^2^)−H selenylation.

In 2022, Choudhury et al. [47] reported a visible-light-mediated C(sp^2^)−H selenation reaction of aminopyrazoles (Scheme 17). The authors chose 5-amino-3-methyl-1*H*-phenylpyrazoles **83** and diphenyl diselenides as model substrates and demonstrated experimentally that the reaction does not require additional metal oxidants; only 12 h of visible-light irradiation in nitrogen at room temperature is required to obtain the desired target products. The reaction also exhibits broad substrate versatility with moderate to good yields and is also suitable for gram-scale synthesis. Finally, the reaction provides important pharmaceutical heterocyclic compounds such as aminopyrazoles, isoxazoles, isothiazoles, and selenium uridines pyrimidines.

**Scheme 18:**
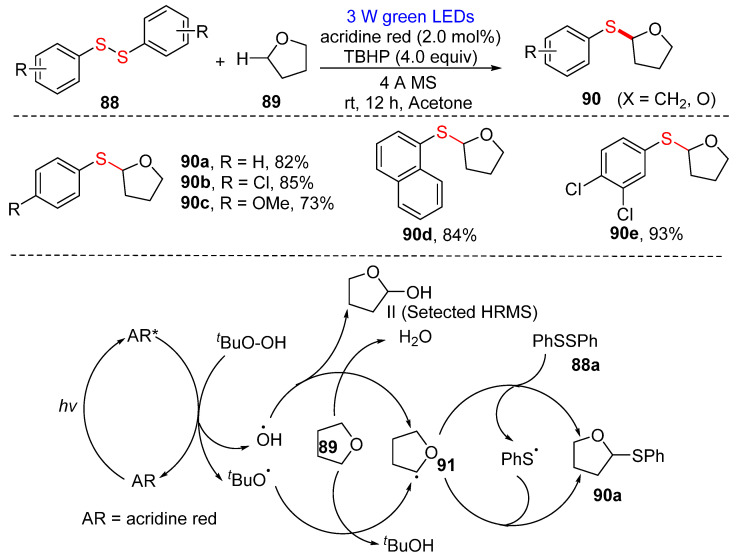
Visible-light-induced direct thiolation of ether with disulfides at *α*-C(sp^3^)−H.

Organosulfur compounds are widely used in medicine, pesticides, new materials, and other fields. Therefore, it is very important to develop new methods for the formation of carbon–sulfur bonds. A reaction strategy for the generation of sulfur radicals from disulfides catalyzed by visible-light has been rapidly developed, and the direct sulfurization of carbon–hydrogen bonds has been widely used. In 2016, Wang’s group [48] developed a simple and effective method for the preparation of *α*-aryl thioethers by direct thiolysis at *α*-C(sp^3^)−H of ethers under visible-light induction using acridine red as a novel photocatalyst (Scheme 18). For this reaction, diphenyldisulfide **88** and tetrahydrofuran **89** were selected as model substrates, acetone as the solvent, and TBHP as the oxidant. When the reaction is irradiated with green light for 12 h at room temperature, the target product **90** can be obtained with a good yield.

According to the free radical experiment results, the authors propose a mechanism, as shown in Scheme 18. First, the AR in the ground state becomes AR* in the excited state under green light irradiation, which is highly reductive. Subsequent interaction with the oxide *^t^*BuOOH (TBHP) gives a hydroxyl radical HO^•^ and a *tert*-butoxy radical *^t^*BuO^•^, while AR* is transformed into the ground state AR. Subsequently, HO^•^ or *^t^*BuO^•^ plucks a hydrogen from tetrahydrofuran (THF) to give the key alkoxy radical intermediate **91**. Intermediate **91** reacts with PhSSPh **88a** to give the desired product 2-(phenylthio)-tetrahydrofuran **90a** and a new radical PhS^•^.

**Scheme 19:**
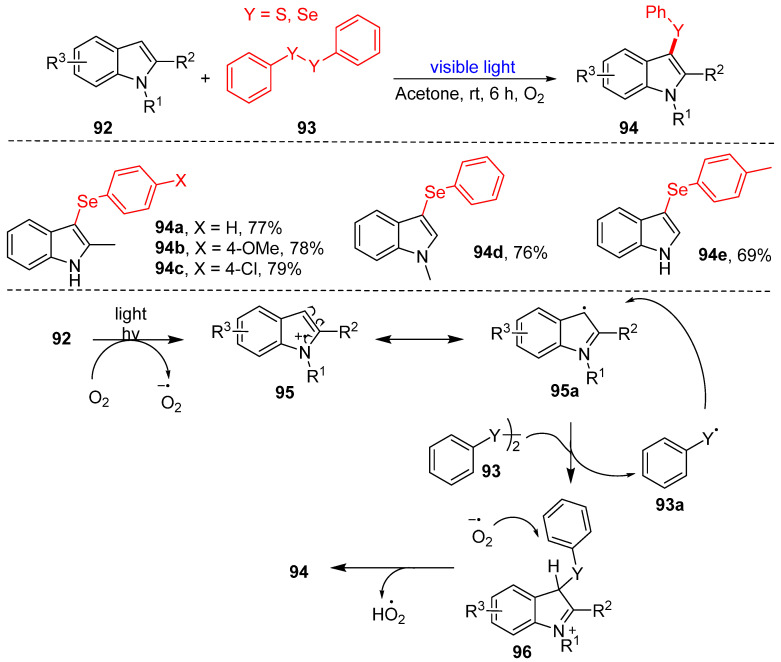
Light mediated C(sp^2^)−H bond chalcogenation (S, Se,) of indoles.

In 2019, the Kumar group disclosed an attractive mild visible-light-induced strategy for the organo-chalcogenylation (S, Se) of indole in an oxygen atmosphere using commercially available substrates in acetone (Scheme 19) [49]. Further, cyclic voltammetry, UV-visible analysis, and electron paramagnetic resonance (EPR) spectroscopic studies have been carried out to establish the mechanism of the light-induced reaction.

**Scheme 20:**
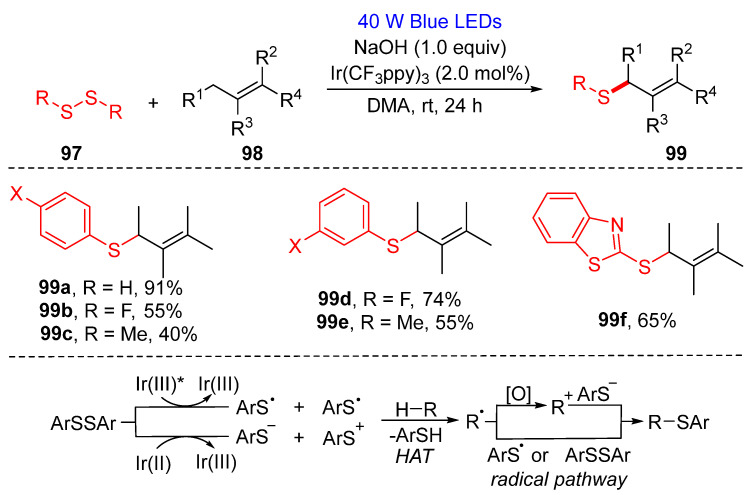
Direct thiolation of allyl C(sp^3^)−H.

Although allyl sulfides are widely used, the direct-catalyzed allyl C(sp^3^)−H sulfidation reaction remains unclear. In 2020, Hong et al. [50] reported a direct photooxidation-catalyzed allyl C(sp^3^)−H sulfidation reaction by visible-light (Scheme 20). The authors initially optimized the reaction conditions using disulfides **97** and 2,3-dimethyl-2-butene **98** as model substrates. The results showed that high yields of allyl sulfides **99** were obtained with 2.0 mol% (CF_3_ppy)_3_ as the catalyst, sodium hydroxide as the oxidant, and dimethylacetamide (DMA) as the solvent under 40 W blue LEDs irradiation for 24 h. In addition, the authors explored the universality of the substrate, and discovered that these reaction conditions were unfortunately not applicable to dialkyl disulfides, with only the corresponding thiols obtained. However, the authors proposed a feasible mechanism for the reaction. First, the sulfur group produced by the interaction of disulfides with an iridium catalyst under light may induce a single-electron transfer of allyl C(sp^3^)−H to obtain allyl radicals, which then undergo radical coupling with the disulfide. In addition, a redox process may occur in the presence of a photocatalyst in which the allyl radical is oxidized to a cationic intermediate, which is ionically coupled to the thioaryl anion to obtain the target product.

**Scheme 21:**
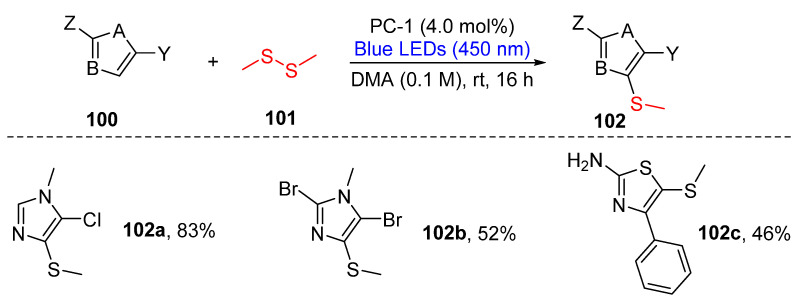
Site-selective thiolation of (multi)halogenated heteroarenes.

Recently, in 2020, the Glorius group published a photocatalytic dithioether reaction for the simple hydrogen methane-thionation of olefins without gaseous and toxic methane-thionate (Scheme 21) [51]. Fortunately, when the reaction uses **100** as a substrate, the reaction requires only the substitution of the thiol coupling with dimethyl disulfide and illumination with a blue light in DMA solvent for 16 h to give the desired product in a very good yield [52,53]. In addition, the reaction is also feasible for tribromoimidazole and 5-chloro-2-aminothiazole, demonstrating the suitability of the methanethiol scheme for a variety of structures.

#### 3.2.2. Direct Selenylation of C−C Bonds

**Scheme 22:**
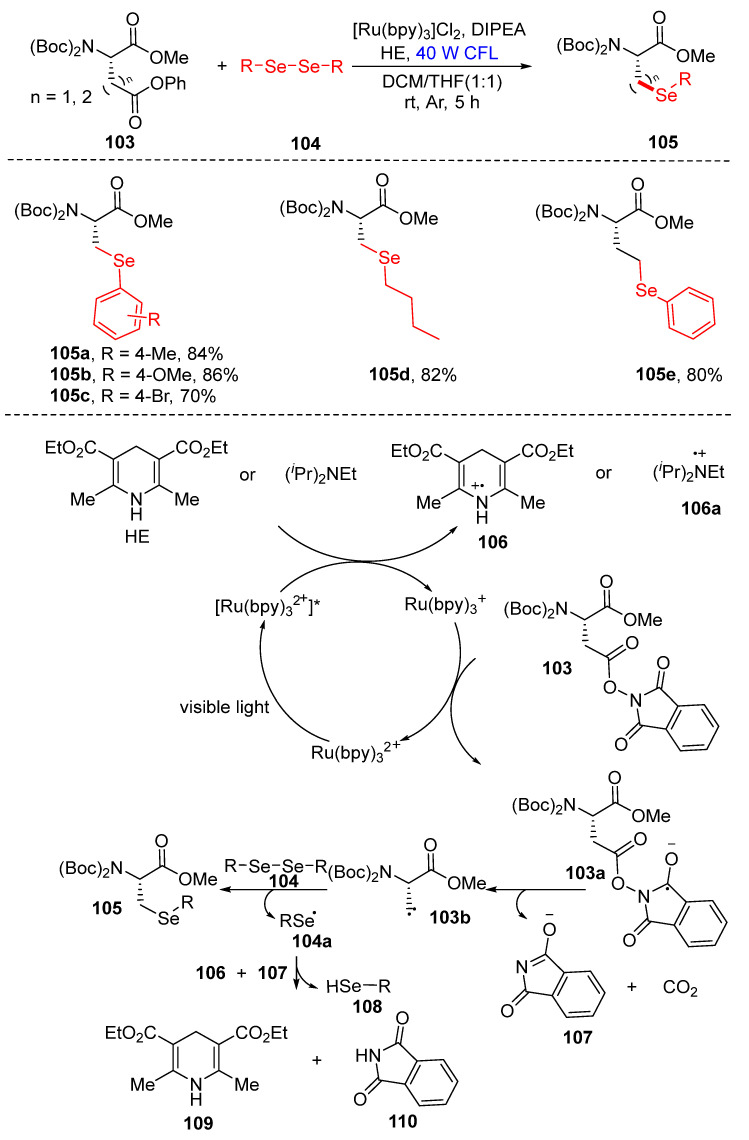
Visible-light photoredox synthesis of chiral *α*-selenoamino acids.

Selenium amino acids are important components for the synthesis of selenoproteins. However, there are few methods for the preparation of selenium amino acids. In 2016, Fu’s group [54] reported a method for the preparation of *α*-selenamine-based compounds using *N*-Bis(Boc)-Asp(oPht)-oMe **103** and diphenyl diselenides **104** as a model substrate (Scheme 22). More importantly, this method enables the synthesis of a series of bioactive and chiral selenoamino acid derivatives. A rational mechanism for the synthesis of chiral *α*-selenoamino acids is proposed in Scheme 22. First, **103**, Ru(bpy)_3_^2+^ is irradiated by visible-light to the excited state [Ru(bpy)_3_^2+^]^*^, which is reduced by diethyl 2,6-dimethyl-1,4-dihydropyridine-3,5-dicarboxylate(HE) or N,N-diisopropylethylamine (DIPEA) to give Ru(bpy)^3+^, where HE or DIPEA became **106** or **106a**. The subsequent interaction of Ru(bpy)^3+^ with **103** gives the radical anion **103a** and the regenerated catalyst Ru(bpy)_3_^2+^, after which **103a** is eliminated to give the benzodicarboximide anion **107** and carbon dioxide to give the radical **103b**. Finally, **104a** and **106** are subjected to free radical coupling to obtain the target product **105**. **104a** interacts with **106** and **107** to give **108**, **109,** and **110**.

**Scheme 23:**
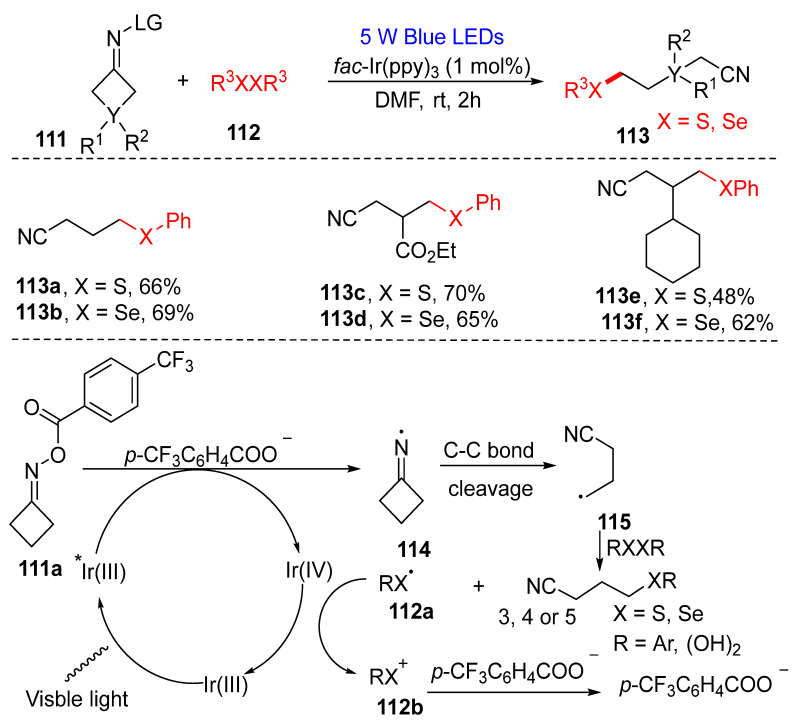
Photochemical C(sp^3^)−Se bond formation through C−C bond cleavage of cyclobutanone oxime esters.

C−H bond activation is one of the most challenging topics in organic synthesis and has received extensive attention from chemists. Finding environmentally friendly catalyst systems for carbon–hydrogen bond activation to construct carbon–selenium bonds has important theoretical and practical value. In 2019, Zhou et al. described the photocatalytic vulcanization, selenization, and boration of cyclosterone oxime esters by the cleavage of C−C bonds initiated by imidamines (Scheme 23) [55]. In this method, using *fac*-Ir(ppy)_3_, diphenyl diselenides **112** and irradiation with blue LEDs several functionalized organoselenides **113** were prepared. Based on the experimental results and previous reports [56,57,58], a plausible mechanism is proposed whereby Ir(III) is converted to the strongly reduced excited state Ir(III)* under visible-light irradiation, reducing **111a** to the imine group **114**. The radical **114** is cleaved by the ring-opening C−C bond to generate the highly reactive cyanoalkyl radical **115**, which is captured by disulfides, diselenides, and diborides, respectively, forming an sp^3^ C−S bond and C−Se bond. The single electron transfer(SET) process of RX^•^ reduction to Ir(IV) produces cation **112b**, which regenerates Ir.

#### 3.2.3. Direct Selenylation/Sulfuration of C−N Bonds

**Scheme 24:**
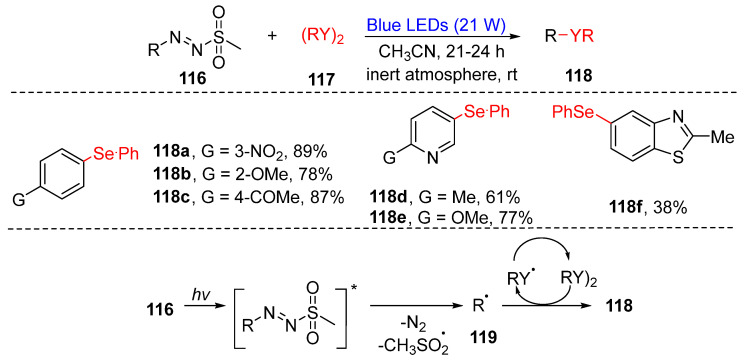
Blue LEDs mediated seleno-substitutions of azosulfone group.

As a common chemical bond, C−N bonds widely exist in organic molecules, and the activation and breaking of C−N bonds play an important role in organic reactions and life chemistry. In 2020, Zhao [59] reported the substitution of azosulfone groups in heterocycles by organoselenide groups driven by blue LEDs. The method is also applicable to the preparation of selenide aromatics and organotelluride products (Scheme 24). The addition of the radical inhibitor TEMPO under standard reactions suggests that the transformation involves a radical process. The authors propose an initial mechanism whereby the (hetero)aryl substrate **116** becomes excited upon irradiation with visible light, followed by the decomposition of the azosulfone group to form the aryl radicals **119**. Aryl radicals **119** with diselenides **117** affords the corresponding selenium-containing compound **118** and the organoselenides RY* group.

**Scheme 25:**
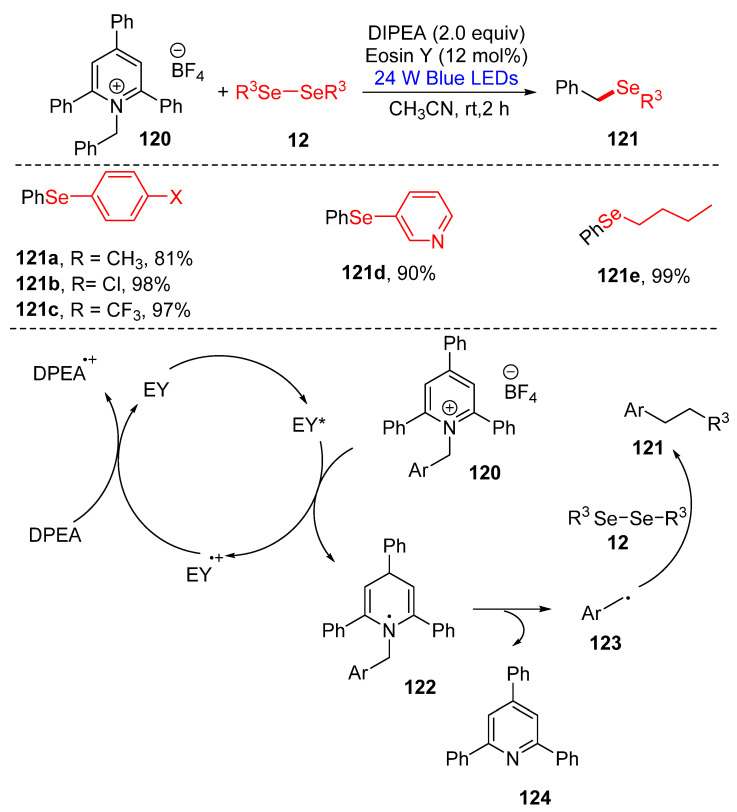
Visible-light-catalyzed synthesis of asymmetric selenides from pyridinium salts.

In the past few years, pyridinium salts have been widely used in organic synthesis as radical precursors due to their ability to reduce single-electron transfer [60]. These salts typically form new C−C, C−N, C−O, and C−B bonds via transition metal catalysis, photocatalysis, and Lewis base activation. However, the formation of carbon–chalcogen bonds by cleavage of pyridinium salts has not been reported to date [61,62,63]. In 2021, Zhao et al. [64] reported a method for the synthesis of asymmetric selenides from alkylpyridinium salts and diselenides under visible-light catalysis (Scheme 25). Radical verification experiments show that the reaction is a radical pathway. First, the authors chose benzyl Katritzky salt **120** and diselenide **12** as model substrates. The selenide product **121** was obtained by reacting with DIPEA as the oxidant and eosin Y as the photocatalyst in an acetonitrile solution at room temperature for 20 h. The reaction is germane to a wide range of applications. First, the excited state of eosin Y (EY*) is reduced by single-electron transfer **120** to yield a dihydropyridine radical **122**, which subsequently decomposes into a benzyl radical **123** and an aromatized pyridine derivative **124**. Single-electron reduction to EY^+^ from DIPEA concurrently regenerates the ground-state eosin Y. Next, benzyl radical **123** reacts with diselenide **12** to give product **121**.

**Scheme 26:**
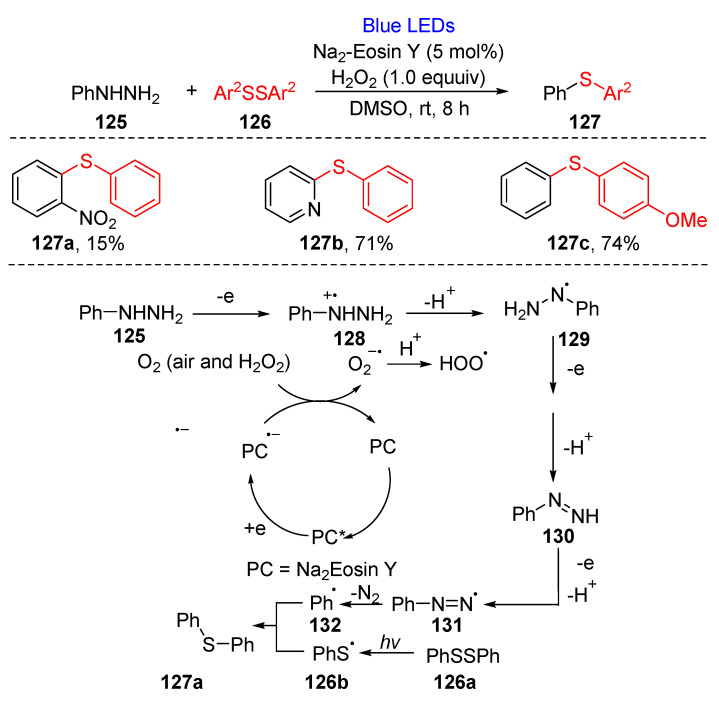
Sulfurization of aryl hydrazines.

In recent years, aryl hydrazides have attracted much attention as electrophilic substitutes in cross-coupling reactions. Aromatics can construct various chemical bonds, including C−C, [65] C−N, [66] C−S, [67] C−Se, [68] and C−P [69] bonds. In particular, aryl hydrazines can act as aryl reactants by releasing nitrogen gas. In 2019, Yu’s group [70] reported the testing of the substrates phenylhydrazine **125** and diphenyl sulfide **126** using the organic dye Na_2_-eosin Y as the catalyst, hydrogen peroxide as the oxidant, and dimethyl sulfoxide (DMSO) as the optimal reaction conditions to yield the expected sulfide product **127** (Scheme 26). Under optimal conditions, they explored a range of visible-light-promoted hydrazine sulfides and showed that substrates with strong electron-absorbing groups were not converted to the corresponding sulfides with high efficiency. This may be due to the low reactivity of the corresponding radical intermediates.

When the sulfidation reaction was carried out with TEMPO or BHT, only a trace amount of **127** was detected based on gas chromatology (GC) analysis. Based on these experimental results and related reports [71], a plausible mechanism is proposed. First, the photocatalyst Na_2_-eosin Y is irradiated to the excited state PC* and then converted to PC- by single-electron transfer, and PC- is oxidized by oxygen (from H_2_O_2_ and air) to provide the state photocatalyst and O^2-·^. At the same time, phenylhydrazine **120** is oxidized to generate the radical cation **12** and the radical cation **128** is deprotonated to provide radical **129**. The radical **129** line is then deprotonated to convert the intermediate **131**. Subsequently, nitrogen is eliminated from **131** to form the phenyl radical **132**. On the other hand, the benzene sulfide radical **126a** is generated by homolytic cleavage of **126** under visible-light irradiation. Finally, the target product, diphenyl sulfide **127a,** is obtained by the radical coupling of **132** and **126b**.

### 3.3. Visible-Light-Enabled Seleno-and Sulfur-Bifunctionalization of Alkenes/Alkynes

Alkenes/alkynes are simple and abundant bulk commodities, and the vicinal difunctionalization of these feedstocks is of great importance in rapidly increasing molecular complexity with a variety of functional groups.

**Scheme 27:**
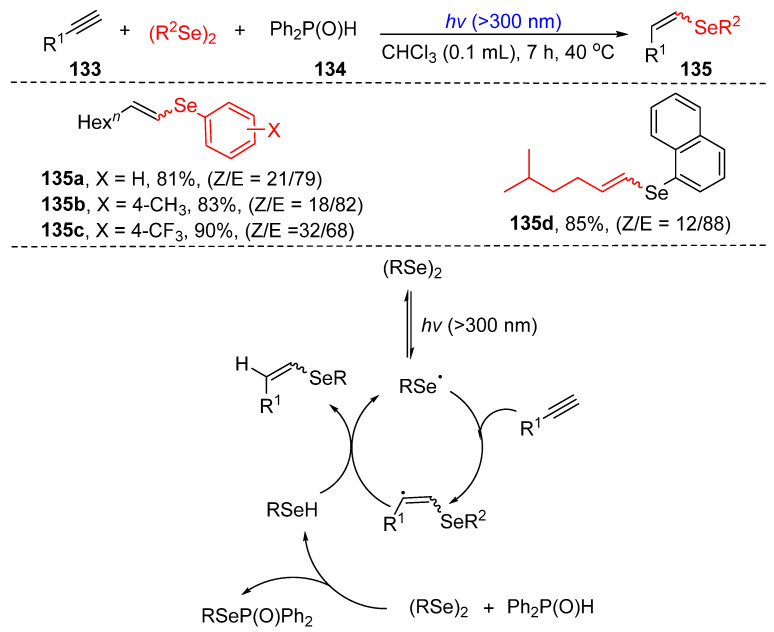
Photochemical regioselective hydroselenylation of terminal alkynes.

In 2013, Ogawa et al. [72] used a diselenide-Ph_2_P(O)H hybridization system to achieve highly regioselective hydroselenation of deactivated terminal alkynes under visible-light irradiation to give vinyl selenide **135**. Based on free radical validation experiments, a reasonable mechanism was proposed, as shown in Scheme 27. First, visible-light irradiation triggers the cleavage of the Se−Se single bond in the diselenides, generating the corresponding selenyl radicals. Next, the attack of the selenyl radicals on the terminal carbon of the olefin leads to the production of the vinyl intermediate. The vinyl radicals are captured by selenol to produce hydroselenate products, while the selenium radicals are regenerated. In addition, Ph_2_P(O)SeR generated as a by-product does not undergo addition reactions with the alkynes. This is in contrast to the addition reaction between the unoxidized Ph_2_PSePh and the alkynes.

**Scheme 28:**
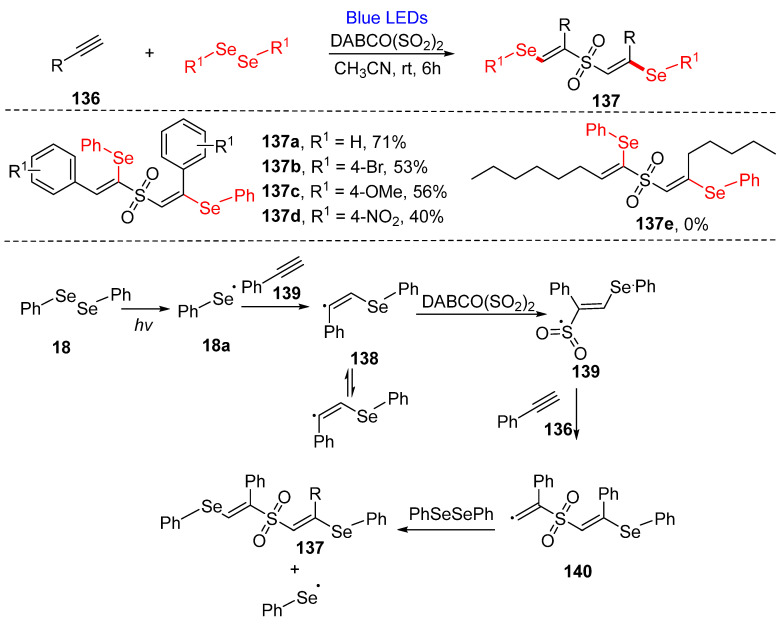
Visible-light-mediated cascading reactions construct C−Se and C−S bonds simultaneously.

In 2019, Chen et al. [73] reported a multi-component cascade reaction of diselenides, alkynes, and sulfur dioxide in the presence of visible light. A novel *β*-sulfonylvinylsilane was prepared by this cascade reaction (Scheme 28). In addition, the conversion reactions of *β*-sulfonylvinylsilanes to 1,4-oxopyrimidine-4,4-dioxides and sulfonylacetylene derivatives were also investigated. The reaction was carried out in a 1,4-diazabicyclo(2,2,2)octane (DABCO)-(SO_2_)_2_ solution with CH_3_CN as the solvent and irradiated under blue light for 6 h. The substituents with halogen, methyl, methoxy, nitro, methoxycarbonyl, and amino groups were detected, respectively. The alkynes and diselenides of alkynes and diselenides showed experimentally that all reactions proceeded smoothly, but the conditions were not applicable to alkylalkynes. The formation of the product was significantly inhibited by the addition of the radical scavenger TEMPO under standard conditions, demonstrating that the transformation involved a radical pathway.

According to previous literature reports [74,75], a feasible reaction mechanism is proposed in Scheme 28. First, under blue light, the diselenide is excited to form selenium radicals. The addition of **136** gives the vinyl radical intermediate **138**, which is then trapped by sulfur dioxide. A sulfonyl radical intermediate **139** is then formed. Subsequent attack on **136** produces the vinyl intermediate **140**, which is captured by disulfide. Finally, product **137** is generated and provides recovery of the selenium radical [76].

**Scheme 29:**
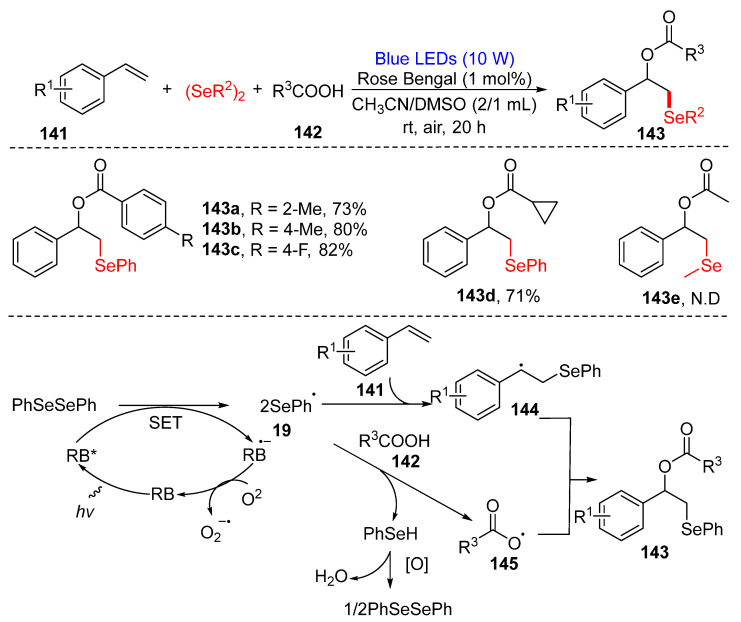
Visible-light-induced difunctionalization of styrenes with a binary system of diaryl diselenides and carboxylic acids.

Difunctionalization of alkenes enables rapid construction of complex molecules with broad applications in organic synthesis. In 2020, Yuan’s group [77] reported a mild and effective strategy for the synthesis of acyloxy selenides via visible-light-induced bifunctionalization of styrene with diaryl diselenides and carboxylic acid dibasic compounds (Scheme 29). The work was carried out under white LEDs (20 W) with 4-methylstyrenes **141**, diphenyl diselenides, and acetic acids **142** as substrates, CH_3_CN as a solvent, and 1.0 mol% RB as the required catalyst. The reaction was carried out for 14 h at room temperature to give the target product **143** with a 58% yield.

Controlled experimental studies confirmed that this process was carried out through a radical chain propagation process. On the basis of controlled and competitive experiments, a reasonable mechanism was depicted, as shown in Scheme 29 [78]. Initially, the rose bengal (RB) produces an excited state under the irradiation of blue LED light. Subsequently, the excited state RB* acquires electrons from diphenyl diselenide, providing RB^•−^ and phenylselenide **19**, which reacts with 4-methylstyrenes **141** to form **144**, and another phenylselenide reacts with acetic acid **142** to form **145** and PhSeH, which is then oxidized by oxygen to PhSeSePh. Finally, the two radicals **144** and **145** react to give the target product **143**.

**Scheme 30:**
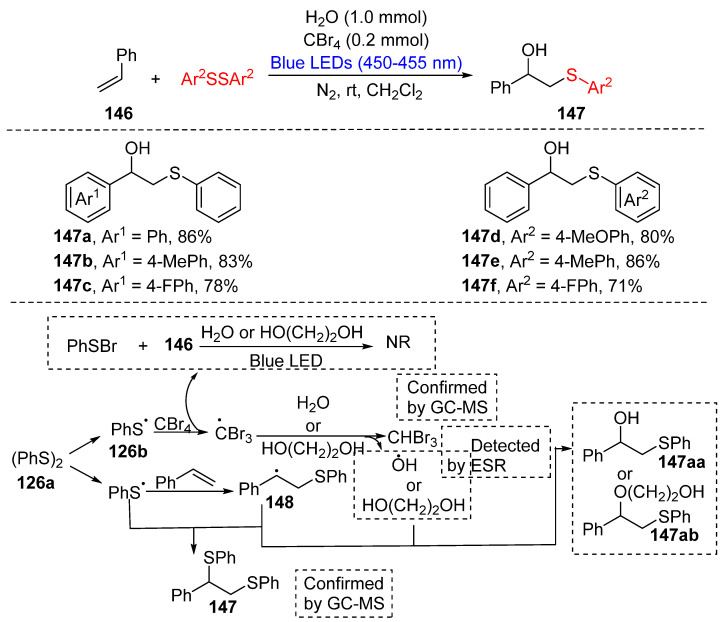
Synthesis of *β*-hydroxysulfide and *β*-alkoxysulfide.

*β*-Oxysulfides are a familiar organic molecule, widely used in organic synthesis precursors and functional compounds [79,80]. This unit is also present in biologically active pharmaceuticals and natural compounds [81]. However, there is no literature on the preparation of *β*-alkoxysulfides using diols as substrates. In 2019, Wang et al. reported a photochemical method for the preparation of bifunctional *β*-oxysulfides without photocatalysts and oxidants (Scheme 30). In this paper, styrene **146** and diphenyl disulfide **47** were used as model substrates to start the study. The desired products, 1-phenyl-2-(phenylthio)ethane-1-ol **147,** were obtained in the presence of dichloromethane (CH_2_Cl_2_), water, CBr_4_, and blue LEDs in the system [82]. Subsequently, the prevalence of the reaction substrates, olefins, and thioethers was explored, and it was found that most of the expected products could be obtained. However, when aliphatic olefins or aliphatic disulfides were used as substrates, none of the expected products were observed.

To elaborate on the possible reaction mechanism, the authors performed a controlled experiment where the difunctionalization was almost suppressed when TEMPO was added to the reaction, which indicates that free radical processes are involved in the reaction. A potential mechanism is outlined in Scheme 30. First, under the irradiation of blue light, the disulfide **126a** is homogenized to generate the sulfur radical **126b**, and then **126b** reacts with CBr_4_ to obtain the tribromomethyl radical, before the hydrogen is extracted from H_2_O or HOCH_2_CH_2_OH to obtain the corresponding hydroxyl group free radicals (^•^OH) or alkoxy groups (^•^OCH_2_CH_2_OH). An addition reaction of **126b** with styrene **146** yields the intermediate **148**. Finally, intermediate **148** with ^•^OH or ^•^OCH_2_CH_2_OH yields the desired products **147aa** or **147ab**.

## 4. Construction of the C(sp)-S Bond

**Scheme 31:**
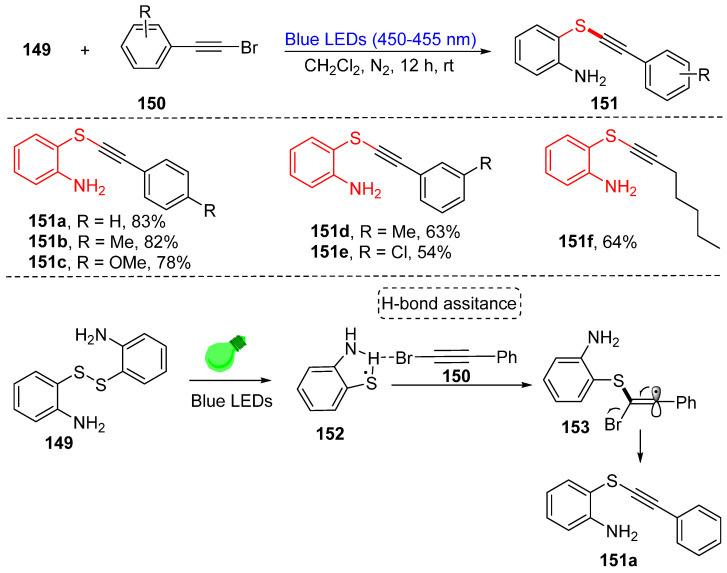
Synthesis of alkynyl sulfides.

The carbon–heteroatom bond formation is used as a bridge for linking organic molecules to access complicated compounds with biological and pharmaceutical activity [83]. Although many synthetic strategies have been established for the C(sp^3^)–S and C(sp^2^)–S bond-forming formation, there are still important challenges for the construction of C(sp)–S bonds, particularly for the achievement of C(sp)–S bond formation [84]. In 2021, Wang et al. reported a strategy for the preparation of alkynyl sulfides using visible-light irradiation (Scheme 31) [85]. First, 2,2′-diaminodiphenyl disulfides **149** and phenylethynyl bromides **150** were chosen as model substrates, irradiated under blue light, and stirred in CH_2_Cl_2_ under an N_2_ atmosphere for 12 h to obtain the desired coupling products **151**. The functional group tolerance of this visible-light-promoted C(sp)−S cross-coupling was investigated under optimized reaction conditions using various brominated alkynes and found to behave well for various brominated alkynes with different aromatic rings and good functional group compatibility.

To clearly illustrate the reaction and establish that the coupling reaction is initiated by disulfide **149** or bromoalkynes **150**, control experiments were performed. The coupling reaction is stopped when the radical scavenger TEMPO is added to the reaction system; this significant inhibition means that the reaction may involve a free radical process. Based on the results of controlled experiments and the relevant literature, the transformation mechanism was proposed in Scheme 31. First, the intermediate product *o*-NH_2_C_6_H_4_S-(**152**) was produced by visible-light-induced homolysis of 2,2’-diaminodiphenyl disulfide (**149**). Then, **152** was added to brominated alkynes **150** to form an intermediate product **153**, which was converted to product **151a** by an elimination reaction.

## 5. Conclusions

In this review, the recent progress in the synthesis and application of disulfide and diselenide as radical reagents with photochemical technology is reviewed, and the reaction scopes, limitations, and mechanisms of some of these reactions are also discussed. From the above discussion, it can be seen that the visible-light-promoted disulfide and diselenide radical reactions provide a stable and robust platform for the efficient construction of various selenium-/sulfur-containing heterocyclic compounds or bifunctional products. The successful examples described in this review convincingly demonstrate the high potential of disulfide and diselenide for drug discovery and applications. Among these synthesis methods, photochemistry and synergistic metal/photoredox catalysis techniques provide a more friendly and sustainable alternative for the application of disulfide and diselenide and illustrate the development prospects of thioether as free radicals.

Although significant progress has been made in this field over the last few decades, several issues and challenges remain in fully developing the potential applications of disulfide and diselenide, which should be further exploited and improved. More catalytic means should be explored in the application of disulfide and diselenide as free radical reagents, especially in terms of electrochemical synthesis methods, because electrochemical strategies have become increasingly powerful tools for the synthesis of organic compounds in recent years. Furthermore, the migration cyclization strategies involving disulfide and diselenide as radical reagents should be established to construct more complex polycyclic compounds, since polycyclic frameworks play an important role in medicinal chemistry and organic materials. In addition, the harmful consequences of metal catalysts and peroxides used in the reaction process cannot be ignored. It is our hope that this paper will encourage more researchers to contribute to this emerging field by developing more environmentally friendly catalytic systems.

## Data Availability

The data presented in this study are available upon request from the authors.
